# Adenosine A_1_-receptor blockade impairs the ability of rat pups to autoresuscitate from primary apnea during repeated exposure to hypoxia

**DOI:** 10.14814/phy2.12458

**Published:** 2015-08-13

**Authors:** James E Fewell, Rongzhi Lun

**Affiliations:** Department of Physiology and Pharmacology, Cumming School of Medicine, University of Calgary, Health Sciences Centre, Alberta Children’s Hospital Research Institute for Child and Maternal HealthCalgary, Alberta, Canada

**Keywords:** Adenosine, autoresuscitation, bradycardia, hypoxia-induced gasping

## Abstract

Failure of gasping to bring about autoresuscitation from hypoxia-induced apnea has been suggested to play a role in sudden unexpected infant death. Little is known, however, about factors that influence the ability of gasping to restore life during severe hypoxia in newborns. Given that adenosine modulates cardiac function during hypoxia-induced apnea and that cardiac dysfunction plays a role in mediating autoresuscitation failure, the present experiments were carried out on 34, 5- to 6-, and 10- to 11-day-old rat pups to investigate their ability to autoresuscitate from hypoxia-induced apnea during repeated exposure to hypoxia after adenosine A_1_-receptor blockade. Each pup was placed into a temperature-controlled chamber regulated to 37 ± 1°C and repeatedly exposed to an anoxic gas mixture (97% N_2_ and 3% CO_2_) until the occurrence of autoresuscitation failure. One group was studied following administration of the selective adenosine A_1_-receptor antagonist 8-Cyclopentyl-1,3,-dipropylxanthine (DPCPX) and one group was studied following vehicle. DPCPX significantly attenuated bradycardia during hypoxia-induced apnea and impaired the ability of both age groups of pups to autoresuscitate during repeated exposure to hypoxia (5–6 days tolerated – vehicle 17 ± 4 vs. DPCPX 10 ± 2 hypoxia exposures [*P* < 0.05]; 10–11 days tolerated – vehicle 10 ± 2 vs. DPCPX 7 ± 2 hypoxia exposures [*P* < 0.05]). Death in all pups resulted from the inability of gasping to restore cardiovascular function during hypoxia-induced apnea although the mechanism of cardiovascular dysfunction/failure was influenced and the occurrence hastened by DPCPX. Thus, our data provide evidence that adenosine acting via adenosine A_1_-receptors enhances the ability of rat pups to tolerate repeated exposure to severe hypoxia during early postnatal maturation.

## Introduction

Newborn mammals are known for their ability to survive a lack of oxygen (Dawes 1968; Adolph 1969). When exposed to a period of unrelenting and progressive hypoxia, newborn rats display a characteristic respiratory response that lasts up to 30 min and consists of tachypnea/hyperpnea, primary apnea, gasping, and secondary or terminal apnea (Gozal et al. 1996; Fewell et al. 2000). After the initial period of tachypnea/hyperpnea, which is mediated by the peripheral chemoreceptors (Lun et al. 2015), primary apnea occurs and may be as short as 10 sec in newborn rats or as long as 18 min in newborn ground squirrels (Adolph 1969; Fewell and Smith 1998). Primary apnea has been viewed as the interval between failure of mechanisms critical to eupneic respiration and the resumption of respiratory activity in the form of gasping (Fung et al. 1994; Wang et al. 1996). Hypoxia-induced gasping occurs when the PaO_2_ decreases to ∼8–10 torr (Guntheroth and Kawabori 1975; Lawson and Thach 1977) and functions as an important survival mechanism with the potential to bring about “self-resuscitation” (Adolph 1969) or “autoresuscitation” (Guntheroth and Kawabori 1975) if it reintroduces oxygen into the gas exchanging region of the lungs and adequate cardiac function has been maintained.

Autoresuscitation by hypoxia-induced gasping requires adequate cardiovascular function to transport oxygen from the lungs to the heart and brain (Gershan et al. 1992; Fewell 2005). Germane to this, electrical and mechanical activities of the heart have been shown to outlast respiratory activity by several minutes when newborns are exposed to a single period of unrelenting and progressive hypoxia (Swann et al. 1954; Dehaan and Field 1959; Cassin et al. 1960); in fact, Swann et al. (1954) have shown that circulatory failure – defined as “that accompanying a blood pressure so low that it can no longer bring reoxygenated blood out of the lungs and thus effect resuscitation” – occurs some 5–10 min after the onset of secondary or terminal apnea in newborn pups. Despite this, autoresuscitation failure occurs in humans and in newborn mice and rats upon repeated exposure to hypoxia (Peiper 1963; Stevens 1965; Gershan et al. 1992; Fewell et al. 2000), and autoresuscitation failure has been documented in human infants dying of SIDS despite the presence of hypoxia-induced gasping (Poets et al. 1999; Sridhar et al. 2003). This suggests a cardiovascular rather than a respiratory root of autoresuscitation failure, which may be related in part to myocardial substrate depletion (Deshpande et al. 1999; Fewell and Zhang 2013).

A prominent feature of the cardiovascular response to severe hypoxia exhibited at the onset of primary apnea – which occurs concurrently with an abrupt reduction in activity and energy consumption of major organs and the switch from aerobic to anaerobic metabolism – is a profound bradycardia (Adolph 1969; Thach et al. 1991; Fewell et al. 2000). Bradycardia during hypoxia-induced primary apnea, which we have shown is primarily mediated in newborn rats by adenosine acting via adenosine-A_1_ receptors, likely serves an important physiological function by reducing cardiac work and thus substrate depletion during oxygen lack (Fewell et al. 2007; Fewell and Zhang 2013). Our current experiments have been carried out to investigate the physiological consequences of tempering the bradycardia during hypoxia-induced primary apnea by testing the hypothesis that prior administration of a selective adenosine-A_1_ receptor antagonist impairs the ability of newborn rats to autoresuscitate during repeated exposure to hypoxia during early postnatal life.

## Materials and Methods

Thirty-four Sprague–Dawley rat pups were studied. Each pup, born by spontaneous vaginal delivery, was housed with its mother and siblings in a Plexiglas cage lined with Aspen-Chip Laboratory Bedding (Northeastern Products, Warrensburg, NY) and kept in a humidity (30–40%) and temperature (25 ± 1°C, the self-selected ambient temperature and thermoneutral temperature of nonpregnant and pregnant rats (Eliason and Fewell 1997)) controlled environmental chamber on a 12:12 h light**–**dark cycle with lights on from 0700. Although 25°C is below the thermoneutral zone of newborn rats (Malik and Fewell 2003), each pup had an opportunity to thermoregulate behaviourally by huddling with its siblings and dam in the nest.

All experimental procedures described herein were carried out in accordance with the “Guide to the Care and Use of Experimental Animals” provided by the Canadian Council on Animal Care, and with the approval of the Animal Care Committee of the University of Calgary.

### Experimental protocol

For an experiment, each 5–6 (*n* = 18) and 10–11 (*n* = 16) day-old rat pup was removed from its mother and siblings and recording devices placed for measurement of cardiovascular and respiratory variables. The pup was then positioned prone in a metabolic chamber regulated to 37 ± mice 1°C into which flowed room air at a rate of 1 L/min for 30 min before receiving a subcutaneous injection of vehicle or 20 mg/kg of the selective adenosine A_1_-receptor antagonist 8-Cyclopentyl-1,3,-dipropylxanthine (DPCPX). After 30-min, the pup was exposed to 97% N_2_ and 3% CO_2_ until hypoxia-induced primary apnea occurred; the chamber gas was then immediately changed back to room air so that room air was inhaled with the first gasp and the ability of the pup to autoresuscitate by hypoxia-induced gasping determined. This procedure was repeated at 5-min intervals until autoresuscitation failure occurred. During an experiment, primary apnea was detected by directly observing respiratory movements on the polygraph tracing.

In order to determine if autoresuscitation had occurred, heart rate and respiratory rate were determined during the last 15-sec of each 5-min period. If heart rate and respiratory rate were greater than 60% of control values, we deemed the autoresuscitation a success. If, however, heart rate and/or respiratory rate during the last 15-sec of each 5-min period were less than 60% of control values, we deemed the autoresuscitation a failure (Jacobi et al. 1991). From experiments carried out on infant mice, Gershan, Jacobi, and Thach (Gershan et al. 1992) have defined three sequential cardiorespiratory stages of a successful autoresuscitation from hypoxia-induced primary apnea. They are stage I – gasping with marked bradycardia; stage II – cardiac resuscitation (with a rapid increase in heart rate to greater than 60% of baseline); and stage III – respiratory resuscitation (with an increase in respiratory rate to greater than 60% of baseline).

### DPCPX and DMSO

DPCPX and DMSO (dimethyl sulfoxide) were obtained from Sigma-Aldrich (Oakville, ON, Canada). Because of the limited solubility of DPCPX in saline, DMSO was used as a solvent for DPCPX and accordingly as vehicle. The rationale for using 20 mg/kg DPCPX in our experiments was that we have previously shown 20 mg/kg to be the smallest dose of the selective adenosine A_1_-receptor antagonist that abolishes the heart rate response to an EC_100_ dose of the selective adenosine A_1_-receptor agonist CPA (N^6^-Cyclopentyl-1,3-dipropylxanthine) (Fewell et al. 2007). As well, it was determined that one dose of 20 mg/kg DPCPX was effective in eliminating the heart rate response to an EC_100_ dose of CPA for up to 90 min in 5–6 and 10–11 day-old rat pups.

### Experimental apparatus

The metabolic chamber used in our experiments consisted of a double-walled plexiglass cylinder (30 cm long – internal diameter 6 cm) into which flowed room air or 97% N_2_ and 3% CO_2._ Chamber ambient temperature was regulated to 37 ± 1°C by circulating water from a temperature controlled bath (Neslab – Endocal Refrigerated Circulating Bath RTE-8DD, Newington, NH) through the space between the walls. The rationale for studying the various age groups of rats at an ambient temperature of 37°C was as follows: Our laboratory has previously determined the self-selected ambient temperature of 5–6 and 10–11 day-old rats, 20- to 30-min after being placed into a 200 cm thermocline with a linear temperature gradient of 25°C–40°C to be 37 ± 1°C (Fewell and Wong 2002). Numerous experiments carried out on newborn as well as older animals provide evidence that when given the opportunity, animals self-select an ambient temperature that is within their thermoneutral zone (Gordon 1985, 1986, 1987; Gordon et al. 1986; Fewell et al. 1997; Malik and Fewell 2003).

### Experimental measurements and calculations

During an experiment, the electrocardiogram, respiratory movements, and chamber CO_2_ levels were recorded on a Model 7 polygraph (Grass Instrument Company, Middleton, WI) at a paper speed of 10 mm/sec. Lead II of the electrocardiogram was recorded from multistranded stainless steel wire electrodes (AS 633; Cooner Wire Company, Chatsworth, CA) placed subcutaneously on the right shoulder, left shoulder, and the left thigh; the electrodes were connected to Model 7HIP5 High Impedance Probes coupled to a Model 7P5 Wide Band EEG A.C. Preamplifiers (Grass Instrument Company). Respiratory movements were recorded from a mercury-in-silicone rubber strain gauge (Model HgPC, D.M. Davis, Inc., Teaneck, NJ) placed around the chest; this highly compliant silicone rubber band does not interfere with breathing. Furthermore, measurement of respiratory activity using this technique, unlike that of a body plethysmograph, allows one to make continuous physiological measurements in the face of rapid and frequent changes in chamber gas composition and concomitant small changes in chamber pressure. The mercury-in-rubber strain gauge was connected to a bridge amplifier (Biomedical Technical Support Center, University of Calgary, Calgary, AB) that was coupled to a Model 7P03 Adapter Panel (Grass Instrument Company).

### Statistical analysis

Statistical analysis was carried out using an ANOVA followed by a Holm-Sidak multiple comparison test. All results are reported as means ± one standard deviation, and *P* < 0.05 was considered to be of statistical significance.

## Results

**Figure 1:**
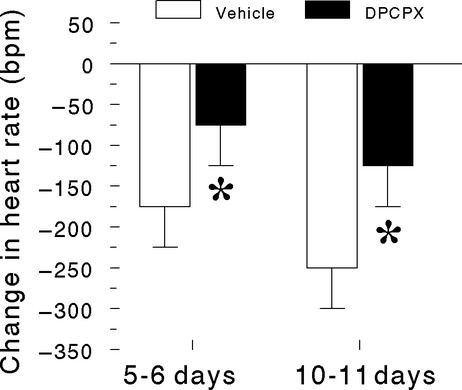
Influence of postnatal age and DPCPX on the change in heart rate from control during hypoxia-induced primary apnea in rat pups. Data are means ± 1 SD; *n* = 9 for vehicle and DPCPX in 5- to 6-day-old pups and *n* = 8 for vehicle and DPCPX 10- to 11-day-old pups. *P < 0.05 vehicle versus DPCPX at a given postnatal age by analysis of variance (ANOVA) followed by a Holm-Sidak multiple comparison test ANOVA. bpm, beats per minute.

Abnormalities in respiratory rhythm (i.e., apnea) or heart rhythm were not observed in any of the pups following administration of vehicle or DPCPX; all animals were awake, active, and “pink” before anoxic gas challenge. Control heart rates were higher in 10–11 day-old pups than in 5–6 day-old pups (*P* < 0.05) but were similar at a given postnatal age in pups that received vehicle and in pups that received DPCPX (5–6 day-old pups, vehicle 396 ± 59 bpm & DPCPX 390 ± 33 bpm [NS]; 10–11 day-old pups, vehicle 442 ± 16 bpm & DPCPX 435 ± 26 bpm, [NS]). As previously reported (Fewell et al. 2007), DPCPX significantly attenuated the heart rate response recorded during hypoxia-induced primary apnea (Fig.1); with regard to the heart rate response, there was an effect of age (*P* < 0.05) and DPCPX (*P* < 0.05) but not an interaction between these independent variables indicating that the effect of DPCPX was not age dependent.

**Figure 2:**
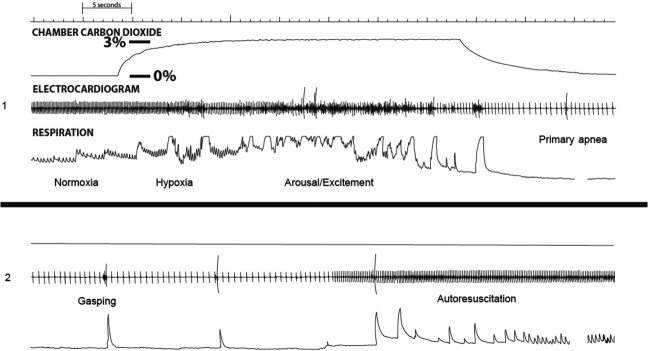
Two continuous segments of a polygraph tracing (i.e., A and B) showing hypoxia-induced gasping bringing about successful autoresuscitation from primary apnea in a 5-day-old rat pup that received vehicle. During exposure to hypoxia there was an initial period of tachypnea/hyperpnea and arousal/excitement that preceded primary apnea and bradycardia; hypoxia-induced gasping was followed by autoresuscitation as evidenced by an increase in heart rate and restoration of a normal respiratory rhythm.

A polygraph tracing showing cardiovascular and respiratory variables recorded during a successful autoresuscitation from hypoxia-induced primary apnea by gasping in a 5 day-old pup that received vehicle is shown in Figure2. All successful autoresuscitations exhibited a similar cardiorespiratory pattern: Initially, there was a period of tachypnea/hyperpnea and arousal/excitement that preceded primary apnea and bradycardia; gasping was followed by an increase in heart rate after 2–3 gasps and then restoration of a normal respiratory pattern. In pups that received vehicle, autoresuscitation failure occurred after 17 ± 4 exposures to hypoxia in 5–6 day-old pups and after 10 ± 2 exposures to hypoxia in 10–11 day-old pups (*P* < 0.05). With regard to the mode of death in 5–6 and 10–11 day-old pups that received vehicle before repeated exposure to hypoxia, all pups exhibited phase I of autoresuscitation (i.e., gasping); death resulted from a lack of sustained phase II of autoresuscitation (i.e., cardiac resuscitation with a rapid increase in heart rate to >60% of baseline) in all pups. Five of nine, 5–6 day-old pups revealed an initial increase in heart rate during gasping before exhibiting terminal atrioventricular dissociation; gasping did not bring about an increase in heart rate in the other 5–6 day-old pups. One of eight, 10–11 day-old pup revealed an initial increase in heart rate during gasping before exhibiting terminal atrioventricular dissociation; gasping did not bring about an increase in heart rate in the other 10–11 day-old pups.

**Figure 3:**
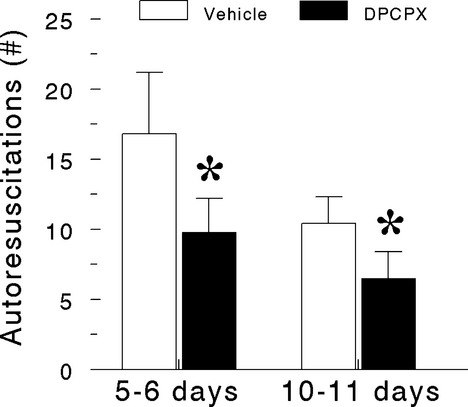
Influence of postnatal age and DPCPX on autoresuscitation from hypoxic-induced primary apnea in rat pups. Data are means ± 1 SD; *n* = 9 for vehicle and DPCPX in 5- to 6-day-old pups and *n* = 8 for vehicle and DPCPX 10- to 11-day-old pups. *P < 0.05 vehicle versus DPCPX at a given postnatal age by analysis of variance followed by a Holm-Sidak multiple comparison test.

**Figure 4:**
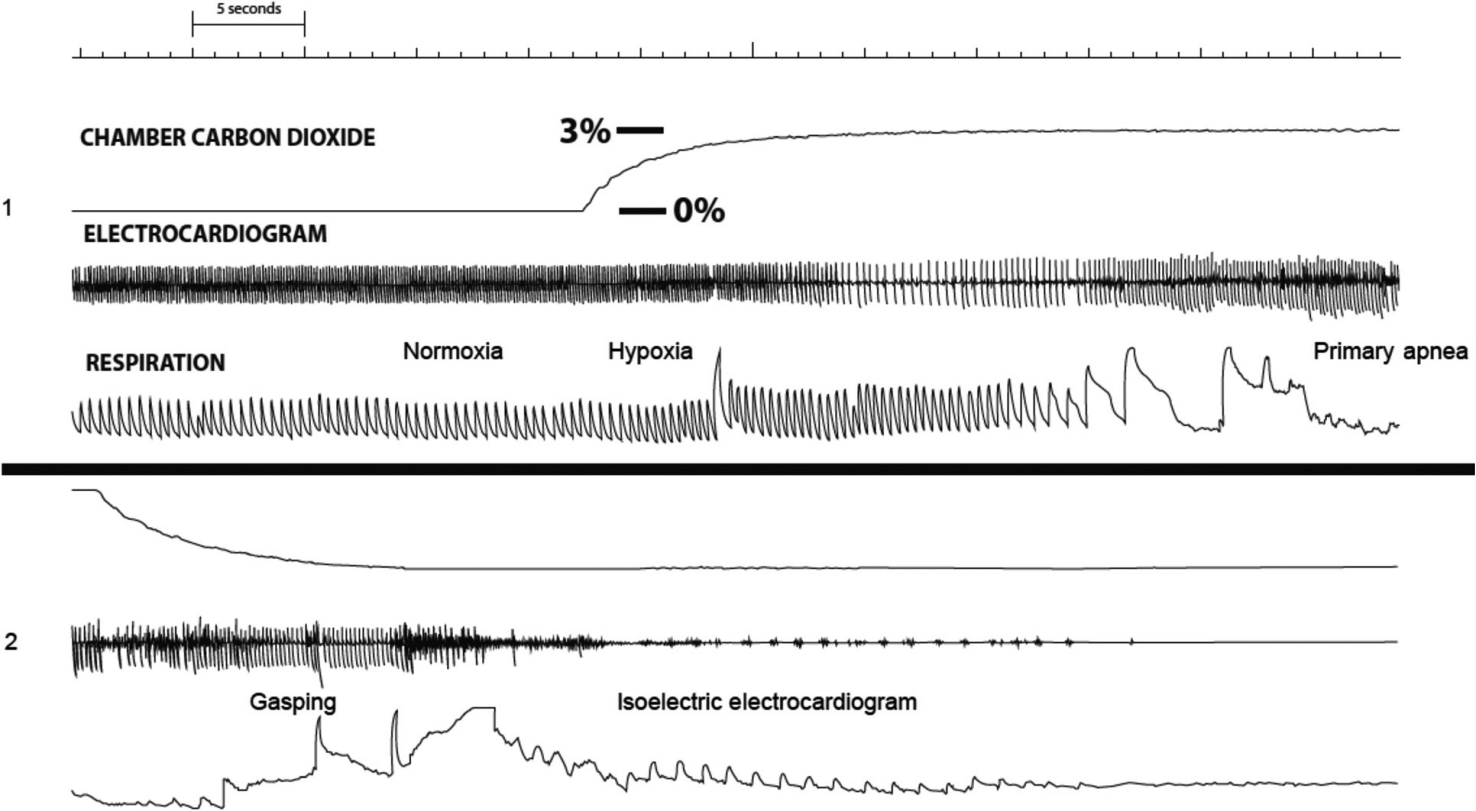
Two continuous segments of a polygraph tracing showing a failed autoresuscitation in a 5-day-old rat pup that received DPCPX. During exposure to hypoxia there was an initial period of tachypnea/hyperpnea but a notable absence of arousal/excitement and profound bradycardia during primary apnea. Note the abrupt disappearance of cardiac electrical activity shortly after the onset of hypoxia-induced gasping despite continued respiratory activity.

Prior administration of DPCPX diminished the ability of 5–6 day-old and 10–11 day-old pups to survive repeated exposure to hypoxia (Fig.3); with regard to number of successful autoresuscitations, there was an effect of age (*P* < 0.05) and DPCPX (*P* < 0.05) but not an interaction between these independent variables indicating that the effect of DPCPX was not age dependent. A polygraph tracing showing cardiovascular and respiratory variables recorded during a failed autoresuscitation from primary apnea despite gasping in a 5 day-old pup that received DPCPX is shown in Figure4. Eight of nine, 5–6 day-old pups and 6 of 8, 10–11 day-old pups displayed this unique and previously unreported pattern of autoresuscitation failure upon repeated exposure to hypoxia. Notable on the polygraph tracing are the lack of a profound bradycardia during hypoxia-induced primary apnea and the complete absence of cardiac electrical activity shortly after the appearance of gasping (i.e., phase I of autoresuscitation). An all-embracing body muscle spasm readily perceived by the experimenter during observation of the rat pup and evident as radiated electromyographic activity on the electrocardiographic tracing preceded the disappearance of cardiac electrical activity.

## Discussion

Our experiments provide new information about factors that influence protective responses of the newborn upon exposure to severe hypoxia. A novel finding was that administration of the selective adenosine-A_1_ receptor antagonist DPCPX impaired the ability of newborn rats to autoresuscitate during repeated exposure to hypoxia. Furthermore, the majority of 5–6 and 10–11 day-old pups displayed a unique and previously unreported pattern of autoresuscitation failure, which consisted of an abrupt disappearance of cardiac electrical activity shortly after the onset of hypoxia-induced gasping. Thus, our data provide evidence that adenosine acting via adenosine A_1_-receptors enhances the ability of rat pups to tolerate repeated exposure to hypoxia during early postnatal maturation. This enhancement likely results from an adenosine-mediated bradycardia during primary apnea, which serves to reduce cardiac work and substrate depletion (e.g., glycogen) during periods of oxygen lack (Fewell et al. 2007; Fewell and Zhang 2013).

Adenosine has long been recognized to have important physiological effects on the cardiovascular system (e.g., chronotrophy, dromotrophy, inotrophy, and coronary vascular resistance) and functions to increase myocardial oxygen delivery and decrease cardiac work during periods of oxygen supply and demand imbalance, thus protecting the heart during hypoxia (Drury and Szent-Gyorgyi 1929; Berne 1980; Belardinelli et al. 1989; Ely and Berne 1992). Its diverse physiological effects are mediated by G-protein coupled receptors designated as A_1_, A_2A_, A_2B_, A_3_ that have been identified based upon agonist and antagonist binding affinities and molecular cloning experiments (Fredholm et al. 1994, 2001). Adenosine principally modulates coronary blood flow via activation of adenosine A_2_-receptors and modulates cardiac electrophysiological activity and contractility via activation of adenosine A_1_-receptors (Belardinelli et al. 1989). Adenosine-A_1_ receptors are present in the fetal rat heart as early as day 14 of their 21 day gestation and functionally coupled to their G protein by day 1 of postnatal life (Cothran et al. 1995; Rivkees 1995; Matherne et al. 1996). We and others have shown that administration of selective adenosine A_1_-receptor agonists (e.g., CPA [N^6^-Cyclopentyl-1,3-dipropylxanthine]) elicits bradycardia in fetal and newborn animals and that high doses cause atrioventricular conduction abnormalities and in some instances cardiac standstill (Smith et al. 1986; Koos and Maeda 2001; Fewell et al. 2007). Furthermore, we have previously shown that adenosine acting via adenosine-A_1_ receptors chiefly mediates the bradycardia observed during hypoxia-induced primary apnea in newborn rats (Fewell et al. 2007).

Administration of the selective adenosine A_1_-receptor antagonist DPCPX blunts the decrease in heart rate normally observed during hypoxia-induced primary apnea (Fig.1) (Fewell et al. 2007) thus disrupting the short-term adaptive mechanism of downregulation of myocardial function to preserve myocardial anaerobic energy substrate and promote survival during hypoxia (Abdel-Aleem et al. 1999). The heart has a high oxygen consumption per tissue mass at rest compared to other organs in the body and extracts ∼75% of the oxygen delivered to it by the coronary circulation (Tune et al. 2004). Thus, an imbalance of myocardial oxygen supply and demand caused by a decrease in arterial oxygen must be met by an increase in coronary blood flow, which is mediated chiefly by adenosine via A_2_-receptors and ATP-dependent K^+^ channels (Berne 1963; Noma 1983). Coronary blood flow to the left ventricle is higher during diastole than during systole because intramuscular blood vessels are compressed and twisted during systole by the contracting heart muscle, which restricts perfusion (Berne and Levy 1972). Since variations in heart rate are accomplished chiefly by shortening or lengthening of diastole, the proportion of time spent in systole and consequently the period of restricted perfusion increases as heart rate increases. Thus, abbreviation of bradycardia during hypoxia-induced primary apnea would be perilous to survival because it not only clamps cardiac work and substrate utilization at relatively high levels but also restricts perfusion of a maximally dilated coronary vascular bed by increasing the period of extravascular compression (Van Citters and Franklin 1969; Berne and Levy 1972; Balaban 2009). These factors in combination likely led to severe cardiac ischemia and acidosis, which resulted in the abrupt cessation of electrical activity observed during repeated exposure to hypoxia in pups that received DPCPX.

We have now described three cardiorespiratory patterns of autoresuscitation failure in rat pups upon repeated exposure to hypoxia, all of which appear to be secondary to cardiovascular dysfunction rather than to a failure of the respiratory system to initiate gasping during severe hypoxia. The first pattern of autoresuscitation failure occurs mainly in younger pups; e.g., 5–6 day-old pups display stage I (i.e., gasping) and stage II (i.e., cardiac resuscitation with a rapid increase in heart rate) of autoresuscitation before the onset of atrioventricular dissociation and the subsequent loss of ventricular depolarization; gasping continues throughout (Fewell et al. 2000). This suggests that heart function and blood pressure are maintained during hypoxia and oxygen introduced into the lungs by gasping is transported to the heart with reoxygenation of atrial pacemaker cells. The subsequent arrhythmia – which appears during the tachycardia of stage II of autoresuscitation – may result from the cardiac effects of adenosine, the myocardial interstitial concentrations of which are well-known to increase during hypoxia (Decking et al. 1988; Moser et al. 1989; Wang et al. 1994; Siaghy et al. 2000).

The second pattern of autoresuscitation failure occurs mainly in older pups; e.g., 10-day and older pups display only stage I (i.e., gasping) of autoresuscitation, as gasping does not induce stage II of autoresuscitation (i.e., cardiac resuscitation with a rapid increase in heart rate) (Fewell et al. 2000). In these pups, cardiac electrical activity in the form of a profound bradycardia – likely of nodal origin – outlasts gasping by many minutes. This suggests that heart mechanical function is not maintained during hypoxia and that despite the introduction of oxygen into the lungs via gasping that oxygen is not transported to the heart for reoxygenation of pacemaker cells. The inability of the heart to maintain cardiac output and blood pressure during hypoxia may result from the depletion of the cardiac metabolic substrate, glycogen – the basal cardiac stores of which are lower in older compared to newborn animals (Shelley 1961; Deshpande et al. 1999). Breakdown of glycogen (i.e., glycolysis) is the major source of energy for anaerobic metabolism in the newborn heart during hypoxia. In support of this postulate, we have recently found and reported that depletion of cardiac glycogen – induced by isoproterenol induced-tachycardia – decreases the number of successful autoresuscitations exhibited by rat pups before autoresuscitation failure during repeated exposure to hypoxia (Fewell and Zhang 2013). On the surface, it would appear that a similar mechanism might be responsible for autoresuscitation failure documented in some human infants dying of SIDS despite the presence of hypoxia-induced gasping (Poets et al. 1999; Sridhar et al. 2003). As early as 1975, Guntheroth and Kawabori (Guntheroth and Kawabori 1975) suggested that autoresuscitation failure occurs in SIDS victims when gasping onset is too late to revive cardiac function.

The third pattern of autoresuscitation failure is described in our current experiments and occurs in both age groups of pups after selective block of adenosine-A_1_ receptors. This pattern differs from the aforementioned two patterns in at least two ways: the first is the lack of a profound bradycardia during hypoxia-induced primary apnea and the second is the abrupt disappearance of cardiac electrical activity shortly after the appearance of gasping. Again, this emphasizes the important participation of adenosine in orchestrating the integrated physiological responses required for successful autoresuscitation during repeated exposure to hypoxia.

In the mature heart of the adult, the sympathetic and parasympathetic divisions of the autonomic nervous system interact to control heart rate. Thus, it is likely that the effect of DPCPX would be different after autonomic innervation of the heart occurs as evidenced by the early work of de Burgh Daly and Scott (de Burgh and Scott 1962; de Burgh and Scott 1963) who showed that the heart rate response to hypoxia stimulation of the carotid body chemoreceptors is dependent upon the respiratory response and mediated by the sympathetic and parasympathetic limbs of the autonomic nervous system. In species which are born relatively immature such as the rat, innervation of the heart is poorly developed at birth and neural control of heart rate is nonexistent or limited (Lipp and Rudolph 1972; Marvin et al. 1980). Sympathetic innervation of the rat heart occurs after birth with functional connections occurring at the end of the first week of postnatal life (Lipp and Rudolph 1972). Functional parasympathetic innervation of the rat heart occurs somewhat earlier than functional sympathetic innervation as Marvin et al. (1980) have shown that vagal stimulation can elicit a decrease in heart rate as early as day 21 of gestation (i.e., term of gestation). Despite this, we have shown that bilateral cervical vagotomy does not influence the heart rate response observed during hypoxia-induced primary apnea in 5–6 day-old rat pups and Adolph (1969) has shown that atropine does not influence the heart rate response to hypoxia in infant rats, 1–11 days after birth (Fewell et al. 2007).

Apnea of prematurity – defined as the cessation of breathing for over 15–20 sec and accompanied by oxygen desaturation and/or bradycardia in neonates with a gestational age less than 37 weeks (Finer et al. 2006; Zhao et al. 2011) – is one of the most frequent pathologies observed in the neonatal intensive care unit, with an incidence of 7% of neonates with a gestational age of 34–35 weeks, 15% of neonates with a gestational age of 32–33 weeks, 54% of neonates with a gestational age of 30–31 weeks, and nearly 100% of neonates with a gestational age of less than 29 weeks or weighing less than 1000 g (Henderson-Smart 1981). Methylxanthines (caffeine, theophylline, and aminophylline) have been used in the treatment of apnea of prematurity since the 1970s (Hascoet et al. 2000; Henderson-Smart and Steer 2001). Whereas theophylline was initially the drug of choice in many neonatal intensive care units, the introduction of a preservative-free parenteral caffeine citrate preparation onto the market in 1999 resulted in a move to caffeine as the drug of choice to this day; caffeine citrate offers the advantages of once daily dosing and a wider therapeutic range with a lower risk of toxicity. Caffeine (1,3,7-trimethylxanthine) acts as an antagonist to endogenous adenosine-A_1_ and adenosine-A_2_ receptor subtypes, and inhibits phosphodiesterase leading to increased levels of cyclic AMP and stimulation of the central nervous system.

Methylxanthine therapy for apnea of prematurity has been shown to have many positive benefits including reductions in apnea and excessive periodic breathing, improved survival without neurodevelopmental disability, shortened duration of oxygen therapy and assisted ventilation, and reduced incidence of bronchopulmonary dysplasia (Shannon et al. 1975; Uauy et al. 1975; Schmidt et al. 2006; Davis et al. 2010); as well methylxanthine therapy has been shown to decrease respiratory abnormalities in term infants at risk for sudden infant death (Kelly and Shannon 1981; Hunt et al. 1983). Cummings et al. (2013) have also recently shown that parenteral administration of caffeine citrate (5 mg/kg) decreases the latency to gasping and facilitates autoresuscitation in serotonin (5-HT) deficient Pet-1^−/−^ mice; these mice normally display prolonged hypoxia-induced primary apnea and impaired autoresuscitation when exposed to episodic asphyxia (Erickson and Sposato 2009; Cummings et al. 2011). The results from our present experiments, however, would allow one to postulate that if methylxanthine therapy were not successful in tempering or terminating apnea via its effect on the respiratory system that the concomitant blockade of cardiac adenosine receptors in the face of severe hypoxia could have a deleterious outcome. We are currently carrying out experiments to determine whether plasma concentrations of caffeine similar to that used clinically to treat apnea of prematurity influence the ability of newborn rats to autoresuscitate from repeated exposure to hypoxia-induced apnea.

In conclusion, our data provide evidence that adenosine acting via adenosine A_1_-receptors enhances the ability of rat pups to tolerate repeated exposure to hypoxia during early postnatal maturation. This enhancement likely results from an adenosine-mediated bradycardia during primary apnea, which serves to reduce cardiac work and substrate depletion (e.g., glycogen) during periods of oxygen lack (Fewell et al. 2007; Fewell and Zhang 2013).
